# Neuroanatomical regions associated with non-progressive dysarthria post-stroke: a systematic review

**DOI:** 10.1186/s12883-022-02877-x

**Published:** 2022-09-16

**Authors:** Marwa Summaka, Salem Hannoun, Hayat Harati, Rama Daoud, Hiba Zein, Elias Estephan, Ibrahim Naim, Zeina Nasser

**Affiliations:** 1 Doctoral School of Sciences and Technology, Lebanese University, Hadath, Lebanon; 2 Medical Imaging Sciences Program, Division of Health Professions, Faculty of Health Sciences, American University of Beirut, Beirut, Lebanon; 3 Faculty of Medical Sciences, Neuroscience Research Center, Lebanese University, Hadath, Lebanon; 4 Faculty of Medical Sciences, Lebanese University, Hadath, Lebanon; 5 Department of Rehabilitation, Health, Rehabilitation, Integration and Research Center (HRIR), Beirut, Lebanon; 6 LBN Univ Montpellier, Montpellier, France

**Keywords:** Non-progressive dysarthria, Stroke, Neuroanatomical regions, Systematic Review

## Abstract

**Background:**

Dysarthria is a common and persisting sequela to stroke. It can have a negative influence on psychological wellbeing, and quality of life. This systematic review aimed to describe and identify the neuroanatomical regions associated with non-progressive dysarthria following stroke.

**Methods:**

A systematic search of PubMed, Ovid Medline, CINAHL, Cochrane, Scopus, and ScienceDirect was conducted to identify all relevant articles published in peer-reviewed journals up to December 2021. Following data extraction, the National Institutes of Health (NIH) quality assessment tools were used to evaluate the methodological quality of the included studies.

**Results:**

Out of 2186 papers found in the literature related to dysarthria post-stroke, 24 met the inclusion criteria. Eligible articles assessed 1150 post-stroke subjects. Out of them, 420 subjects had dysarthria from isolated lesions. Regarding dysarthric subjects with ischemic strokes, 153 sustained supratentorial infarctions, while 267 had infratentorial infarctions. The majority had pontine infarctions (*n* = 142), followed by infarctions in the corona radiata (*n* = 104), and the cerebellum (*n* = 64).

**Conclusion:**

This systematic review is the first step toward establishing a neuroanatomical model of dysarthria throughout the whole brain. Our findings have many implications for clinical practice and provide a framework for implementing guidelines for early detection and management of dysarthria post-stroke.

**Supplementary Information:**

The online version contains supplementary material available at 10.1186/s12883-022-02877-x.

## Background

Non-progressive dysarthria is a motor speech disorder characterized by weakness, incoordination, slowness of the speech musculature, and speech intelligibility [1, 2]. It is induced by non-progressive diseases of the central nervous system, such as traumatic brain injury (TBI) and stroke [3, 4]. Non-progressive dysarthria is estimated to occur in 20–42% of stroke survivors and two-thirds of all subjects with their first-ever ischemic stroke [5, 6]. It leads to negative social and emotional consequences [1, 7–9] that can last for months after a stroke. Taking into consideration the high incidence of dysarthria post-stroke [5] and the associated negative sequelae, it is crucial to identify the stroke occurrence early on and to prevent its complications.

Computed tomography (CT) is the gold standard diagnostic tool for stroke due to its affordability, accessibility, and rapid image acquisition [10], whereas magnetic resonance imaging (MRI) is also regarded as a valuable and complementary diagnostic tool [11]. Recent neuroimaging studies showed that dysarthria post-stroke is related to lesions involved in speech-related areas, which include the primary motor cortex, the lateral premotor cortex as well as the prefrontal cortices, the supplementary motor area [12, 13], the corona radiata, the internal capsule, the striatocapsular area, the midbrain, the pons, the medulla, and the cerebellum [13–19]. These findings have the potential to provide a summary of the neuroanatomical predictors of dysarthria post-stroke stroke.

To our knowledge, there has been no review investigating the occurrence of dysarthria due to single or multiple lesions, and its anatomical location in subjects with stroke. A synthesis of the literature on the presentation of dysarthria in adults with stroke, in addition to information on brain lesions associated with the development of this impairment, will help healthcare providers and speech therapists implement early assessments and interventions. The aim of this systematic review is, therefore, to describe and identify the neuroanatomical regions associated with non-progressive dysarthria following stroke.

## Methods

### Protocol and registration

The current systematic review was performed following the Preferred Reporting Items for Systematic reviews and Meta-Analyses guidelines (PRISMA) [20]. A statement of ethics was not required. The study protocol was registered on PROSPERO (registration number: CRD42022310796). The initial protocol included the performance of a meta-analysis. However, the planned meta-analysis was not performed because most of the studies did not report common outcome and our objective was restricted to identifying the neuroanatomical brain regions associated with non-progressive dysarthria among subjects with stroke.

### Information sources and search strategy

A systematic literature search was carried out on the following medical electronic databases: PubMed, Ovid Medline, CINAHL, Cochrane, Scopus, and ScienceDirect. The review addressed studies published in peer-reviewed journals up to the 31^st^ of December 2021. The databases were searched using the following keywords: “stroke”, “cerebrovascular accident”, “dysarthria”, “neuroimaging”, “magnetic resonance imaging”, “positron Emission Tomography”, and “Computed Tomography”. Boolean Operators (AND, OR) were used to combine the keywords. The search strategy was as follows: dysarthria and (stroke or “cerebrovascular accident”) and (neuroimaging or “magnetic resonance imaging” or “positron Emission Tomography” or “computed tomography”). For PubMed, Ovid Medline, CINAHL, and Cochrane databases, all the keywords were used as exploded medical subject headings (MeSH) except for “cerebrovascular accident” which was used as a keyword. The MeSH term used for computed tomography was “Tomography, X-Ray Computed”. For CINAHL database, the MeSh “neuroradiology” corresponded to “neuroimaging”. For Scopus and ScienceDirect databases, the keywords were used as free-text. The search was restricted to articles published in English and no other limits or filters were used. Furthermore, reference lists from eligible articles were hand-searched to identify more relevant papers for inclusion.

### Eligibility criteria

Eligible studies used neuroimaging techniques including MRI, positron emission tomography, or CT to identify brain lesions associated with the presence or absence of dysarthria post-stroke. Eligible studies included adults (> 18 years) with acquired non-progressive dysarthria post-stroke. The accepted study designs were (1) cross-sectional studies, (2) cohort studies, (3) randomized controlled trials, (4) and case series with > 10 participants. Consequently, excluded studies were (1) narrative or systematic reviews, (2) case reports, (3) correspondence, (4) editorial or expert opinions, (5) methodological articles, (5) conference abstracts, (6) studies involving children and adolescents, (7) studies that did not report dysarthria outcomes according to brain region, and (8) studies on transient dysarthria or dysarthria induced by medications.

### Selection and data collection process

Search and identification of eligible studies were performed independently by two reviewers (MS and HZ). All the retrieved references were imported into EndNote X8 software and duplicates were removed. The two reviewers (HZ and RD) screened titles and abstracts to select eligible papers. Any disagreements were resolved by discussion and cross-checking the papers.

Data extraction was conducted independently by MS and RD using Microsoft Office Excel. For each full-text paper, detailed information was collected on basic study information (last author’s name, publication year, and country of study), study design and sample size, presence of a control group, participant characteristics (mean age, gender), as well as the neuroimaging technique used to investigate the presence/absence of dysarthria, site, side of the lesion, type of stroke, and the main findings.

### Synthesis methods

Within each paper, we identified the location of the lesion for each subject with stroke, whether it was multiple or isolated and the presence or absence of dysarthria. The findings were synthesized according to neuroanatomical brain regions. The relative frequency of dysarthria within studies was calculated and the findings were presented accordingly. Data collected by the two reviewers (MS and RD) were compared and discrepancies were resolved by consensus. The distribution of the neuroanatomical locations associated with dysarthria post-stroke were reported for each study individually (see additional file 1).

### Methodological quality assessment

The National Institutes of Health (NIH) quality assessment tools were used to assess the quality of all the eligible studies (available at: https://www.nhlbi.nih.gov/health-topics/studyquality-assessment-tools). The quality assessment was performed independently by two reviewers (MS and ZN); discrepancies were resolved by discussion and consensus. The NIH quality assessment tools were developed to assess the quality of observational studies including cross-sectional, retrospective observational, and case series studies [21]. It is commonly used and recommended nowadays [21]. The quality of cross-sectional and retrospective observational studies is rated based on fourteen items, while case series studies are evaluated based on nine items (see Additional file 2). The ratings on the different items are obtained by using yes or no responses. For each “yes” response, 1 point is awarded for the evaluated study. The quality of each study is decided on the total score obtained. Cross-sectional and retrospective observational studies that score ≥ 11 points and case series studies that score ≥ 7 points are considered of “good” quality. Furthermore, cross-sectional and retrospective observational studies of 6 to 10 points and case series studies of 4 to 6 points are considered of “fair” quality. Studies of lower scores are defined as having “poor” quality.

## Results

### Study selection

**Fig. 1:**
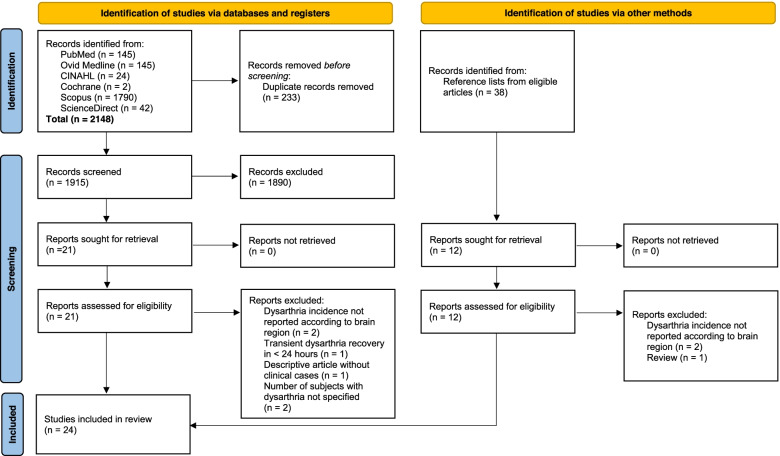
Flow chart of study selection strategy

Figure 1 illustrates the flowchart of the selection process. The literature search retrieved a total of 2186 studies. 2148 papers were identified from databases, while 38 papers were identified from reference lists. After removing duplicate records (*n* = 233), a total of 1953 studies were assessed for eligibility. Screening uncovered 33 papers for full-text review. Following the review process, nine papers did not meet the eligibility criteria [22–30], while 24 papers met the inclusion criteria and were included in the systematic review.

### Study characteristics

Table 1 reports the characteristics of the 24 included studies (published between 1992 and 2017). Both ischemic (*n* = 22) and hemorrhagic (*n* = 2) strokes were analyzed. The studies presented seven countries, namely Germany [17, 31–34], Switzerland [19, 35, 36], Turkey [16, 37, 38], Korea [39, 40], the United States of America [41], Japan [42–45], and South Korea [18, 46–49]. The total number of assessed subjects was 1150 adults (aged 18 years and above), out of whom 577 subjects had dysarthria. Gender ratio of 2:1 (male to female) was reported, with males representing 64.09% (*n* = 737) and females 34.34% (*n* = 395). Gender was not addressed in one of the studies, representing 1.57% of subjects (*n* = 18) [33].

**Table 1:** Study characteristics for all subjects

**Study**	**Country**	**Sample size (*n* = 1150)**	**Enrollment period**^b^ **years;months**	**Mean age (range)**	**Gender males** **n**	**Stroke type**	**Neuroimaging technique used**	**Days to initial imaging**	**Method of dysarthria assessment**^j^	**Days to dysarthria assessment**^j^
Ackermann et al. [17]	Germany	12	NS^c^	55.8 (34–75)	8	Ischemic	MRI^e^ and/or CT^f^	≤ 7	Neurophonetic test battery	≤ 7
Barth et al. [35]	Switzerland	34	3;0	67 (24–87)	25	Ischemic	MRI	3–7	Clinical	NS
Bassetti et al. [36]	Switzerland	36	3;0	64 (20–80)	26	Ischemic	MRI and CT	≤ 7	Clinical	NS
Beckmann et al. [37]	Turkey	64	4;0	64.2 (29–87)	30	Ischemic	MRI and CT	≤ 5	Clinical	NS
Canbaz et al. [16]	Turkey	55	0;3	65.5 (18–97)	36	Ischemic	MRI and/or CT	NS	Clinical	≤ 3
Chung et al. [39]	Korea	215	3;0	57.4 (27–90)	133	Hemorrhagic	CT	NS	Clinical	NS
Erdemoglu and Duman [38]	Turkey	21	NS	62 (31–85)	14	Ischemic	CT	≤ 3	Clinical	NS
Kase et al. [41]	United States of America	66	5;0	61(18–88)	46	Ischemic	MRI or CT	NS	Clinical	NS
Kataoka et al. [42]	Japan	49	11;0	67.2 ± 6.^7d^	31	Ischemic	MRI	7–14	Clinical	3–7
Kim et al. [49]	South Korea	26	8;0	58.6 (34–89)	14	Hemorrhagic	MRI or CT	≤ 21	Clinical	On admission
Kim [46]	South Korea	13	5;0	56 (33–72)	9	Ischemic	MRI and/or CT	≤ 5^ g^	Clinical	NS
Kim [47]	South Korea	130	8;0	57 (28–84)	90	Ischemic	MRI	NS	Clinical	NS
Kim and Kim [40]	South Korea, Canada	40	6;9	65 (47–81)	23	Ischemic	MRI	≤ 10	Clinical	NS
Kim et al. [18]	South Korea	37	3;4	61 (36–85)	19	Ischemic	MRI and/or CT	NS	Clinical	NS
Min et al. [48]	South Korea	31	1;3	59 (42–80)	24	Ischemic	MRI	NS	Clinical	NS
Okuda et al. [43]	Japan	12	9;0	67.8 (49–81)	10	Ischemic	MRI	10–54	Clinical	NS
Schmahmann et al. [50]	US	25	8;0	61 (32–82)	15	Ischemic	MRI or CT	NS	Clinical	NS
Tanaka et al. [44]	Japan	31	3;0	68.1 ± 11.6	19	Ischemic	MRI	< 1^ h^	Clinical	On admission
Tohgi et al. [45]	Japan	64	NS	61.9 (NS)	47	Ischemic	MRI and CT	CT: on admission MRI: ≤ 30	Clinical	NS
Urban et al. [31]	Germany	68	5;0	65.2 (34–86)	43	Ischemic	MRI or CT	≤ 3	Neurophonetic test battery	≤ 3
Urban et al. [32]	Germany	18	NS	64 (45–82)	13	Ischemic	MRI and/or CT	NS	Neurophonetic test battery	≤ 5
Urban et al. [33]	Germany	18	NS	NS	NS	Ischemic	MRI and/or CT	≤ 1	Neurophonetic test battery	≤ 7
Urban et al. [34]	Germany	62	3;2	64.7 (34–87)	44	Ischemic	MRI and/or CT	NS	Neurophonetic test battery	≤ 3
Vuilleumier et al. [19]	Switzerland	23^a^	3;1	62.5 (30–85)	18	Ischemic	MRI	≤ 16^i^	Clinical	1–16

^a^Five subjects with lower brainstem lesions were excluded from the initial sample: one subject had medullary infarction associated with parietal aneurysm and the other four subjects had unclassified infarctions

^b^The length of data collection period

^c^Not specified

^d^mean ± standard deviation

^e^Magnetic resonance imaging

^f^Computed Tomography

^g^All subjects were examined within 5 days except for 3 patients

^h^Median onset-to-imaging time

^i^All subjects were examined within 16 days (mean of 6 days) except for 2 patients

^j^Starting from stroke onset

Dysarthria assessment was performed through clinical evaluation (*n* = 19/24) [16, 18, 19, 35–50], conducted either by a neurologist (*n* = 15/19) [16, 18, 19, 36–42, 44–47, 50], or a psychiatrist (*n* = 1/19) [49], or unspecified (*n* = 3/19) [35, 43, 48]. Dysarthria assessment was also done through a formal assessment tool, performed by experienced speech and language therapists (*n* = 5/24) [17, 31–34].

As for the neuroimaging techniques used for lesion analyses, eight studies reported using MRI [19, 35, 40, 42–44, 47, 48], two studies used CT [38, 39], while the remaining fourteen studies used either MRI or CT or a combination of both [16–18, 31–34, 36, 37, 41, 45, 46, 49, 50] (Table 1).

### Quality assessment

Table 2 summarizes the results of the methodological quality assessment of the eligible studies using NIH quality assessment tools. The present systematic review included 8 cross-sectional studies, 6 retrospective observational studies, and 10 case series. The cross-sectional studies were awarded seven to ten points out of fourteen, retrospective observational studies obtained seven to eight points, while case series studies received five to seven points out of nine. Based on the reviewer’s quality criteria on the NIH quality assessment tools, all cross-sectional and retrospective observational studies were considered of “fair” quality. Case series studies had “fair to good” quality (Table 2).

**Table 2:** Quality assessment of included studies using the National Institutes of Health (NIH) quality assessment tools

Study	Study design	Quality assessment score	Quality rating
Ackermann et al. [17]	Case series	6/9	Fair
Barth et al. [35]	Cross-sectional	7/14	Fair
Bassetti et al. [36]	Retrospective observational	7/14	Fair
Beckmann et al. [37]	Cross-sectional	9/14	Fair
Canbaz et al. [16]	Cross-sectional	9/14	Fair
Chung et al. [39]	Retrospective observational	8/14	Fair
Erdemoglu and Duman [38]	Case series	5/9	Fair
Kase et al. [41]	Retrospective observational	7/14	Fair
Kataoka et al. [42]	Cross-sectional	10/14	Fair
Kim et al. [49]	Retrospective observational	8/14	Fair
Kim [46]	Case series	6/9	Fair
Kim [47]	Retrospective observational	8/14	Fair
Kim and Kim [40]	Cross-sectional	9/14	Fair
Kim et al. [18]	Case series	6/9	Fair
Min et al. [48]	Case series	6/9	Fair
Okuda et al. [43]	Case series	6/9	Fair
Schmahmann et al. [50]	Case series	5/9	Fair
Tanaka et al. [44]	Retrospective observational	8/14	Fair
Tohgi et al. [45]	Cross-sectional	9/14	Fair
Urban et al. [31]	Cross-sectional	9/14	Fair
Urban et al. [32]	Case series	6/9	Fair
Urban et al. [33]	Case series	7/9	Good
Urban et al. [34]	Cross-sectional	10/14	Fair
Vuilleumier et al. [19]	Case series	6/9	Fair

### Neuroanatomical regions of interest

Table 3 shows the neuroanatomical regions of stroke and the frequency of dysarthria in all subjects. Twenty-four articles reported the frequency of dysarthria in 1150 subjects with stroke; 909 subjects had ischemic strokes while 241 had hemorrhagic strokes. All articles evaluated the presence/absence of dysarthria according to discrete neuroanatomical regions of interest. All subjects are reported to have circumscribed lesions in multiple or single discrete brain region. Lesions restricted to a single brain region or area are considered isolated lesions. Eight of the 24 studies were conducted on subjects all diagnosed with dysarthria due to stroke to inspect associated brain regions [16, 31–34, 43, 46, 49]. However, the remaining studies were conducted on subjects with ischemic/hemorrhagic strokes to investigate and associate clinical symptoms, including dysarthria, with brain lesions [17–19, 35–42, 44, 45, 47, 48, 50].

**Table 3:** Main findings related to the distribution of brain regions associated with dysarthria post-stroke

**Ischemic Stroke**	**Variables**	**Relative frequency of dysarthria within studies for all subject (*n* = 909)**	**Subjects with dysarthria after lesions in multiple/isolated regions (*n* = 577)**	**Subjects with dysarthria after isolated lesions (*n* = 420)**
	**Brain region**	**n/N (%)**	**n**	**n**
	**Supratentorial involvement**	***n* = 270**	***n* = 235**	***n* = 153**
	Motor cortex	20/20 (100)	20	20
	Middle cerebral artery cortex	9/9 (100)	9	9
	Internal capsule	18/18 (100)	18	18
	Corona Radiata	105/140 (75)	105	104
	Corona radiata and/or internal capsule	43/43 (100)	43	0
	Striatocapsular area	38/38 (100)	38	0
	Thalamus	1/1 (100)	1	1
	Basal ganglia	1/1 (100)	1	1
	**Infratentorial involvement**	***n* = 639**	***n* = 342**	***n* = 267**
	Cerebellum	96/208 (46.15)	96	64
	Midbrain	28/48 (58.33)	28	28
	Pons	183/230 (79.57)	183	142
	Medulla oblongata	35/153 (22.88)	35	33
**Hemorrhagic stroke**	**Variables**	**Relative frequency of dysarthria within studies for all subject (*n* = 241)**	**Subjects with dysarthria after lesions in multiple/isolated regions (*n* = 17)**	**Subjects with dysarthria after isolated lesions (*n* = 14)**
	**Brain region**		**n**	**n**
	**Supratentorial involvement**	***n* = 241**	***n* = 17**	***n* = 14**
	Striatocapsular area	3/215 (1.39)	3	0
	Basal ganglia	14/26 (53.85)	14	14

Lesions were counted once for each subject; % percentage

### All Subjects with stroke

Out of 909 subjects with ischemic strokes, 29.70% (*n* = 270) had sustained supratentorial strokes. Infarctions were located in the motor cortex (*n* = 20), middle cerebral artery cortex (*n* = 9), internal capsule (*n* = 18), corona radiata (*n* = 140), corona radiata and/or internal capsule (*n* = 43), striatocapsular area (*n* = 38), thalamus (*n* = 1), and basal ganglia (*n* = 1). The motor cortex involved the cortical or subcortical motor area [46] and the lower part of the primary motor cortex [16, 31, 32, 34]. The middle cerebral artery territory included the motor cortex and the corona radiata [16, 31]. Internal capsule lesions included the genu, the posterior limb, and the anterior limb [16, 31, 32]. Corona radiata lesions were documented in six studies [16, 31, 32, 37, 45, 46], among which two specified lesions of the centrum semiovale area [31, 37]. Two articles identified lesions in the corona radiata and/or internal capsule [43, 44]. The Striatocapsular area involved lesions in the basal ganglia and the internal capsule [16, 31, 34, 46]. One article specified isolated lesions in the basal ganglia, specifically in the caudate nucleus [16]. Finally, hemorrhagic strokes involved the striatocapsular area (*n* = 215) and the basal ganglia (*n* = 26).

The remaining 639 subjects (70.30%) sustained infratentorial strokes located in the cerebellum (*n* = 208), midbrain (*n* = 48), pons (*n* = 230), and medulla oblongata (*n* = 153). Cerebellar infarctions were detected in nine articles [16, 17, 31, 33–35, 38, 41, 48]. The distribution of the cerebellar infarctions in terms of arterial territories was obtained from all the articles except for one (*n* = 31) (48). Subjects with cerebellar infarctions (75.95%, 158/208) had infarctions restricted to one cerebellar territory. The majority (*n* = 91) had superior cerebellar artery (SCA) infarctions, 64 subjects had posterior cerebellar artery (PICA) infarctions, and three subjects had anterior inferior cerebellar artery (AICA) infarctions. As for the midbrain region, it extended rostrocaudally from the region just below the lower thalamus to the region just above the midbrain-pontine junction [31, 40, 42]. Pontine infarctions were located at the base of the pons [16, 18, 31, 34, 36, 42, 45, 46, 50]; subjects with tegmental or cerebellar involvement were not counted as having isolated pontine lesions. Two articles reported lesions in the medulla oblongata including lateral, medial, and dorsal medullary regions [19, 47].

Regarding the laterality of lesion, it was described for 1038 subjects, where 448 sustained left-side lesions, 362 sustained right-side lesions, and 36 sustained bilateral lesions. 192 subjects were reported to have unilateral lesions without specifying the side. However, four studies did not specify laterality data [38, 40, 48, 50].

### Subjects with dysarthria post-stroke

**Fig. 2:**
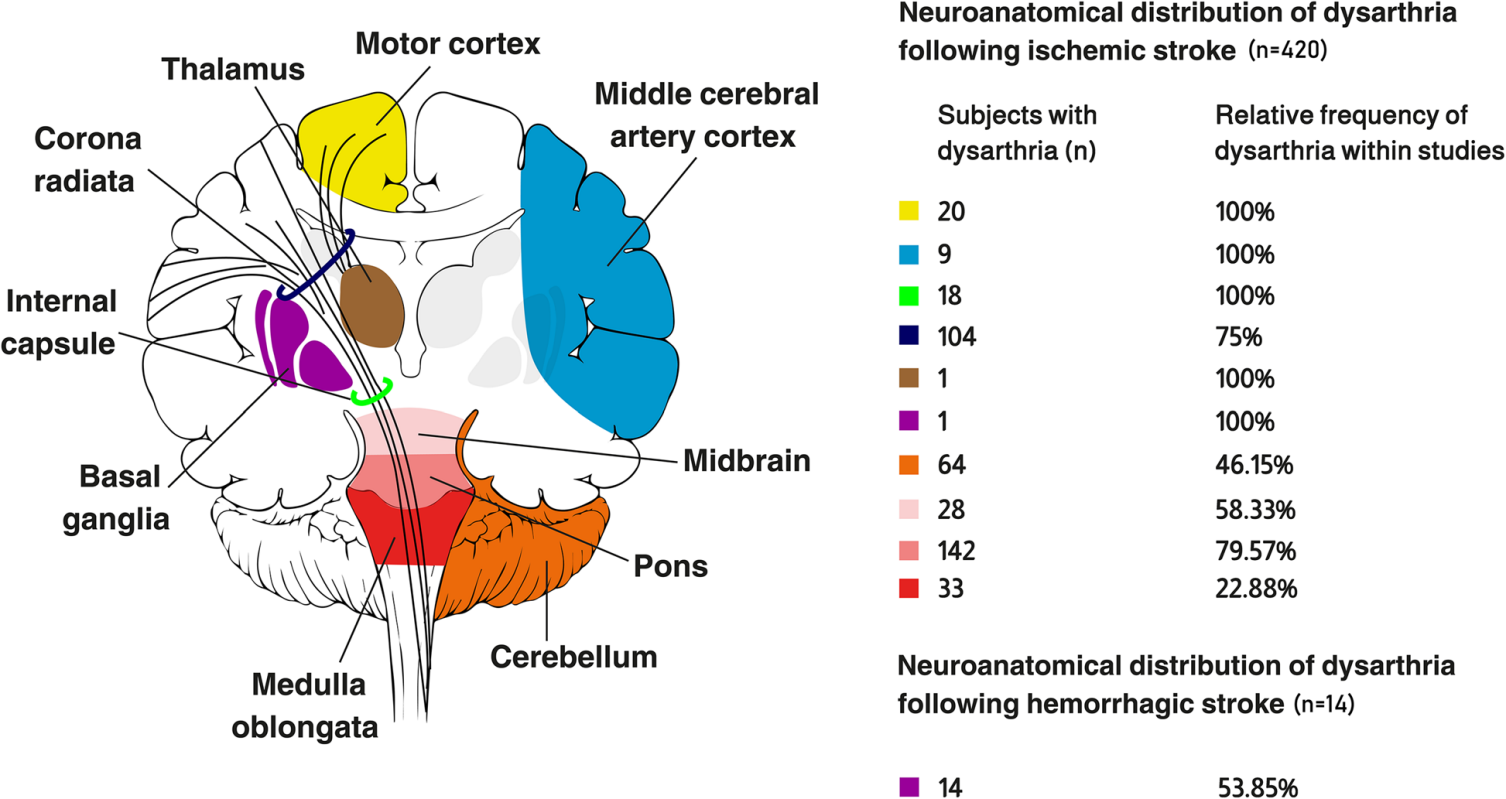
Graphical presentation of the neuroanatomical locations associated with dysarthria post-stroke in subjects with isolated lesions

The frequency of dysarthria post-stroke was obtained from isolated brain lesions (Table 3). The relative frequency of dysarthria within studies (n/N) was calculated for each brain region. Within the sample of 684 subjects with isolated lesions from ischemic strokes, 420 presented dysarthria. Of them, 153 sustained supratentorial infarctions, while 267 had infratentorial infarctions. For supratentorial brain regions including the motor cortex, middle cerebral artery cortex, internal capsule, thalamus, and basal ganglia, the relative frequency of dysarthria within the included studies was 100% since these studies were conducted exclusively on subjects with dysarthria. On the other hand, the relative frequency of dysarthria in subjects following corona radiata infarctions was 75%. 104 subjects with dysarthria had infarctions located in the corona radiata, while 20 subjects had motor cortex infarctions. Regarding infratentorial regions, 142 subjects with dysarthria had pontine infarctions and 64 had cerebellar infarctions. For subjects with hemorrhagic strokes in the striatocapsular area or basal ganglia (*n* = 238), 14 presented dysarthria. Figure 2 shows the graphical distribution of the neuroanatomical locations associated with dysarthria post-stroke in subjects with isolated lesions. Individual-level data for laterality of lesion in subjects with dysarthria from isolated lesions were present for 270 subjects. Left-side lesions occurred in 167 subjects, right-side lesions in 93 subjects, and bilateral in 10.

Eight studies included subjects with pure dysarthria having no additional neurological symptoms, with a total of 46 subjects [16, 31, 34, 40, 43–46]. 20 subjects with pure dysarthria had isolated infarctions. The location of isolated lesions was reported for 19 subjects and was cited in the corona radiata (*n* = 8; 6 left-sided and 2 unclassified laterality), the midbrain (*n* = 1, left-sided), and the pons (*n* = 10, 1 right-sided, and 9 unclassified laterality). On the other hand, 23 subjects with pure dysarthria had infarctions located in the corona radiata and/or internal capsule (*n* = 22; 12 left-sided, 2 right-sided, and 8 bilateral) and striatocapsular area (*n* = 1; right-sided). Also, 3 subjects with pure dysarthria had lesions in the corona radiata and/or internal capsule combined with pontine infarcts. (Table 3).

## Discussion

The current study aimed to identify brain regions associated with dysarthria post-stroke. Overall, 24 stroke-related articles were reviewed in this systematic review (22 on ischemic strokes and two on hemorrhagic strokes). A total number of 1150 subjects with stroke were assessed. All articles reported individual-level data for dysarthria frequency according to neuroanatomical brain lesions, as well as the neuroimaging technique used to investigate brain lesions. and identified the subjects who received neuroimaging scans. However, discrepancies in the neuroimaging modality and its scheduling post-stroke were identified. Similarly, the method and timing of dysarthria assessment differed across studies. Also, inconsistencies in the individual-level data for age, gender, and laterality of lesion were reported, thereby limiting the possibility of assessing their influence on the frequency of dysarthria.

According to the included studies, the majority of supratentorial infarctions were restricted to the corona radiata (*n* = 104), followed by the motor cortex (*n* = 20), and the internal capsule (*n* = 18). Isolated brain stem infarctions were distributed across the pons (*n* = 142), the midbrain (*n* = 28), and the medulla oblongata (*n* = 33). When compared with previous findings, extracerebellar lesions causing dysarthria were reported along the pyramidal tract [31, 32, 51, 52]. The majority of the pyramidal tract axons originate from the primary motor cortex [53], with the corticobulbar tract emerging from the lower part of the precentral gyrus [54]. The corticobulbar tract passes into the corona radiata through the centrum semiovale to be arranged and compressed into the internal capsule. They travel through the rostral capsule in the anterior half of the posterior limb, then shift to the caudal capsule in the posterior half of the posterior limb reaching the brain stem [55, 56]. The proposed model explains the distribution of brain lesions associated with dysarthria since they are generally located along the pyramidal tract. This is supported by the fact that the majority of subjects with dysarthria presented with motor weaknesses or pyramidal signs, as the neural basis of dysarthria follows the course of the pyramidal tract. As for subjects with isolated or pure dysarthria, it is reported that they presented small-sized infarcts mainly relating to classical lacunar syndromes [31, 44, 46].

Dysarthria due to cerebellar lesions has been frequently reported after SCA infarctions [16, 17, 31, 33–35, 38, 41]. In isolated cerebellar infarctions restricted to the SCA territory, dysarthria ranged from 50 to 100% of cases [16, 17, 31, 33–35, 38, 41]. Barth et al. and Urban et al. demonstrated that dysarthria occurred after PICA and AICA infarctions [31, 33, 35]. Similarly, Amarenco et al. reported that four subjects with dysarthria had AICA infarctions at autopsy [57]. However, dysarthria associated with PICA or AICA infarctions was always found to have brainstem involvement [31, 33, 35, 57] which frequently occurs in cerebellar infarctions due to the shared vascular system [57–59]. Nonetheless, dysarthria is also frequent following brainstem infarctions [16, 18, 19, 31, 34, 36, 40, 42, 45–47, 50], thereby, it is hard to determine whether dysarthria in subjects with combined lesions is due to cerebellar infarction, brainstem involvement, or both.

Information on lesion laterality was available for 64.29% (270/420) of subjects with dysarthria from isolated lesions. The majority (61.85%) had left-sided lesions, 34.44% had right-sided lesions, and 3.70% had bilateral lesions. Several studies have stated that dysarthria is more frequently caused by left-side lesions [31, 32, 34, 44–46, 49]. Urban et al. reported that 81.5% of subjects with dysarthria from extracerebellar infarctions had left-sided lesions, while 18.5% had right-sided lesions [31]. Another study conducted by Urban et al. demonstrated that 88.7% of extracerebellar infarctions leading to dysarthria were located in the left hemisphere and that dysarthria severity was more expressed in left-side lesions despite the lesion site [34]. In contrast, Canbaz et al. reported that 51.9% of extracerebellar infarctions leading to dysarthria were found to be located in the right hemisphere [16], whereas Alexander et al. and Wildgruber et al. reported that right-sided lesions do not cause dysarthria [60, 61]. This difference might be justified by the lesions of a common descending tract, such as the corticobulbar fibers reaching the articulatory muscles [32, 51, 52]. Urban et al. proposed that a lesion of the corticolingual pathway is crucial to the pathophysiology of dysarthria from stroke, thus, it is suggested that this might be related to a more dominant descending pathway originating from the left motor area [31].

As for isolated cerebellar infarction, the lesion side responsible for dysarthria is still debatable. In two different studies, Urban et al. reported that dysarthria was more frequently associated with right-sided cerebellar infarctions [31, 33]. On the other hand, in the study of Ackermann et al. lesions were equally distributed on the left and the right side in ten subjects and bilateral in two [17]. A right-side dominance was proposed by Urban et al., but it has not yet been proven [33].

Included studies in this systematic review were observational studies of cross-sectional, retrospective, and case series design. The quality assessment revealed some methodological limitations. Indeed, only five studies [19, 39, 44, 47, 48] reported the blindness of investigators to the subjects’ clinical information. Furthermore, most studies used informal clinical assessment of dysarthria, whereas only five [17, 31–34] used standardized assessments but without reporting their psychometric properties. Despite these methodological flaws, all studies reported consecutive enrollment and selection of subjects with stroke, reflecting homogeneity of sampling across the studies. Besides, all studies identified lesions sites and frequency of dysarthria accordingly. To the best of our knowledge, this is the first review that systematically synthesized data from observational studies to describe the neuroanatomical regions associated with dysarthria post-stroke. This review contributed to capturing the combined picture of several individual study results. The findings of this study will be helpful for the early identification of dysarthria post-strokes, especially those localized in brain regions of the highest risk. Consequently, speech and language therapy assessments will be conducted earlier to deliver indications for appropriate intervention in subjects with stroke.

The current review had several limitations. Although the search strategy was as comprehensive as possible, it might have inadvertently missed some relevant papers and failed to identify the grey literature. In addition, there were discrepancies in the frequency of dysarthria post-stroke across the different articles. This may be related to the absence of consensus for a standardized evaluation and differences in the timing of dysarthria assessment. Another factor that might have influenced the results is the small sample sizes that may restrict the capture of true frequency. Moreover, individual studies showed inconsistencies in the reported variables and individual-level data. Most of the eligible studies did not report measures of effect size, which hindered the comparison of results across studies. It is also worth noting that Middle Eastern countries displayed a clear deficiency in this research topic, which might be related to limited research on speech therapy-related topics and lack of data collection [62].

The findings of the present study generate interesting questions and future directions for forthcoming studies. Further research is needed to compare the findings of this review with parallel bodies of literature identifying the frequency of dysarthria after other diseases of the central nervous system. Although not the purpose of the current review, few eligible studies assessed the evolution of dysarthria post-stroke. Future work is encouraged to study the recovery patterns in subjects with strokes while studying the influence of age, gender, location, and side of the lesion. In parallel, the neuroanatomical regions associated with the fast recovery of dysarthria and those resulting in persistent symptoms may be pinpointed.

## Conclusion

The current systematic review of 24 observational studies suggests that brain lesions associated with dysarthria post-stroke were located along the supratentorial and infratentorial regions. It is the first step toward establishing a neuroanatomical model of dysarthria throughout the whole brain. Our findings have many implications for clinical practice and are of high significance to the field of speech and language therapy and provide a framework for the early detection of dysarthria post-stroke. Subsequently, speech and language therapists should be attentive to the neuroanatomical regions associated with dysarthria to be able to conduct earlier screening in subjects with the highest risk.

## Supplementary Information

**Additional file 1.**

**Additional file 2.**

## Data Availability

Data are available from the corresponding authors upon reasonable request.

## Abbreviations

NIH: National Institutes of Health
TBI: Traumatic brain injury
CT: Computed tomography
MRI: Magnetic resonance imaging
PRISMA: Preferred Reporting Items for Systematic reviews and Meta-Analyses
SCA: Superior cerebellar artery
PICA: Posterior cerebellar artery
AICA: Anterior inferior cerebellar artery
