# Supplementary material for: Neuroanatomical regions associated with non-progressive dysarthria post-stroke: a systematic review
Source: BMC Neurol. 2022 Sep 16;22:353. doi: 10.1186/s12883-022-02877-x (PMC9479301; doi:10.1186/s12883-022-02877-x)
Supplement: Supplementary file 1 — Additional file 1. [file 12883_2022_2877_MOESM1_ESM.docx]

**Neuroanatomical regions associated with non-progressive dysarthria post-stroke: A systematic review**

Marwa Summaka^1^, Salem Hannoun^2^, Hayat Harati^3^, Rama Daoud^4^, Hiba Zein^5^, Elias Estephan^3,6^, Ibrahim Naim^5^, Zeina Nasser^3*^

^1^Doctoral School of Sciences and Technology, Lebanese University, Hadath, Lebanon; ^2^Medical Imaging Sciences Program, Division of Health Professions, Faculty of Health Sciences, American University of Beirut, Beirut, Lebanon; ^3^Faculty of Medical Sciences, Neuroscience Research Center, Lebanese University, Hadath, Lebanon; ^4^Faculty of Medical Sciences, Lebanese University, Hadath, Lebanon ; ^5^Department of Rehabilitation, Health, Rehabilitation, Integration and Research Center (HRIR), Beirut, Lebanon; ^6^LBN Univ Montpellier, Montpellier, France.

Corresponding author: Zeina Nasser ^3^* MSc, PhD, Faculty of Medical Sciences, Neuroscience Research Center, Lebanese University, Hadath, Lebanon. Email: [z.nasser@ul.edu.lb](mailto:z.nasser@ul.edu.lb); ORCID: Zeinanasser2020; Telephone: 961 70950261-Fax: 961 1610920

Table 1. Dysarthria frequency according to brain regions in all subjects post-stroke

| **Ischemic stroke** | **Study** | **Brain region** | **Subjects with stroke (n=909)** | **Dysarthria frequency after multiple/isolated regions n/N** | **Dysarthria frequency after isolated lesions n/N** |
| --- | --- | --- | --- | --- | --- |
|  | **Ackermann et al. [1]** | Cerebellum | **12** | **4/12** | **3/6** |
|  | **Barth et al. [2]** | Cerebellum | **34** | **13/34** | **13/20** |
|  | **Bassetti et al. [3]** | Pons | **36** | **22/36** | **5/9** |
|  | **Beckmann et al. [4]** | Corona Radiata | **64** | **34/64** | **34/64** |
|  | **Canbaz et al. [5]** | Motor cortex | 1 | 1/1 | 1/1 |
|  |  | Middle cerebral artery cortex | 8 | 8/8 | 8/8 |
|  |  | Internal capsule | 5 | 5/5 | 5/5 |
|  |  | Corona Radiata | 20 | 20/20 | 20/20 |
|  |  | Striatocapsular area | 2 | 2/2 | 0 |
|  |  | Thalamus | 1 | 1/1 | 1/1 |
|  |  | Basal ganglia | 1 | 1/1 | 1/1 |
|  |  | Cerebellum | 3 | 3/3 | 3/3 |
|  |  | Pons | 14 | 14/14 | 14/14 |
|  | **Total** |  | **55** | **55/55** | **53/53** |
|  | **Erdemoglu and Duman [6]** | Cerebellum | **21** | **14/21** | **14/21** |
|  | **Kase et al. [7]** | Cerebellum | **66** | **9/66** | **9/54** |
|  | **Kataoka et al. [8]** | Midbrain | 7 | 5/7 | 5/7 |
|  |  | Pons | 42 | 41/42 | 27/27 |
|  | **Total** |  | **49** | **46/49** | **32/34** |
|  | **Kim [9]** | Motor cortex | 2 | 2/2 | 2/2 |
|  |  | Corona radiata | 5 | 5/5 | 4/4 |
|  |  | Striatocapsular area | 3 | 3/3 | 0 |
|  |  | Pons | 3 | 3/3 | 3/3 |
|  | **Total** |  | **13** | **13/13** | **9/9** |
|  | **Kim [10]** | Medulla oblongata | **130** | **28/130** | **28/130** |
|  | **Kim and Kim [11]** | Midbrain | **40** | **22/40** | **22/40** |
|  | **Kim et al. [12]** | Pons | **37** | **11/37** | **11/37** |
|  | **Min et al. [13]** | Cerebellum | **31** | **12/31** | **-** |
|  | **Okuda et al. [14]** | Corona radiata and/or internal capsule | **12** | **12/12** | **0** |
|  | **Schmahmann et al. [15]** | Pons | **25** | **23/25** | **14/16** |
|  | **Tanaka et al. [16]** | Corona radiata and/or internal capsule | **31** | **31/31** | **0** |
|  | **Tohgi et al. [17]** | Corona Radiata | 28 | 23/28 | 23/28 |
|  |  | Pons | 36 | 32/36 | 32/36 |
|  | **Total** |  | **64** | **55/64** | **55/64** |
|  | **Urban et al. [18]** | Motor cortex | 4 | 4/4 | 4/4 |
|  |  | Middle cerebral artery | 1 | 1/1 | 1/1 |
|  |  | Internal capsule | 6 | 6/6 | 6/6 |
|  |  | Corona radiata | 16 | 16/16 | 16/16 |
|  |  | Striatocapsular area | 4 | 4/4 | 0 |
|  |  | Cerebellum | 14 | 14/14 | 6/6 |
|  |  | Midbrain | 1 | 1/1 | 1/1 |
|  |  | Pons | 22 | 22/22 | 21/21 |
|  | **Total** |  | **68** | **68/68** | **55/55** |
|  | **Urban et al. [19]** | Motor cortex | 4 | 4/4 | 4/4 |
|  |  | Internal capsule | 7 | 7/7 | 7/7 |
|  |  | Corona radiata | 7 | 7/7 | 7/7 |
|  | **Total** |  | **18** | **18/18** | **18/18** |
|  | **Urban et al. [20]** | Cerebellum | **18** | **18/18** | **7/7** |
|  | **Urban et al. [21]** | Motor cortex | 9 | 9/9 | 9/9 |
|  |  | Striatocapsular area | 29 | 29/29 | 0 |
|  |  | Cerebellum | 9 | 9/9 | 9/9 |
|  |  | Pons | 15 | 15/15 | 15/15 |
|  | **Total** |  | **62** | **62/62** | **40/40** |
|  | **Vuilleumier et al. [22]** | Medulla oblongata | **23** | **7/23** | **5/14** |
| **Hemorrhagic stroke** | **Study** | **Brain Region** | **Subjects with stroke (n=241)** | **Dysarthria frequency after multiple/isolated regions n/N** | **Dysarthria frequency after isolated lesions n/N** |
|  | **Chung et al. [23]** | Striatocapsular area | **215** | **3/215** | **0** |
|  | **Kim et al. [24]** | Basal ganglia | **26** | **14/26** | **14/26** |
| ^a^ Not specified | | | | | |
|  |  |  |  |  |  |

References

1. Ackermann, H., et al., Speech deficits in ischaemic cerebellar lesions. Journal of neurology, 1992. **239**(4): p. 223-227.

2. Barth, A., J. Bogousslavsky, and F. Regli, The clinical and topographic spectrum of cerebellar infarcts: a clinical—magnetic resonance imaging correlation study. Annals of Neurology: Official Journal of the American Neurological Association and the Child Neurology Society, 1993. **33**(5): p. 451-456.

3. Bassetti, C., et al., Isolated infarcts of the pons. Neurology, 1996. **46**(1): p. 165-175.

4. Beckmann, Y.Y., et al., Clinical and Radiologic Correlations of Small and Large Centrum Ovale Infarcts. Journal of Stroke and Cerebrovascular Diseases, 2010. **19**(3): p. 220-224.

5. Canbaz, D.H., et al., Dysarthria in Acute Ischemic Stroke: Localization and Prognosis. Journal of Neurological Sciences, 2010. **27**(1).

6. Erdemoglu, A. and T. Duman, Superior cerebellar artery territory stroke. Acta neurologica scandinavica, 1998. **98**(4): p. 283-287.

7. Kase, C., et al., Cerebellar infarction. Clinical and anatomic observations in 66 cases. Stroke, 1993. **24**(1): p. 76-83.

8. Kataoka, S., et al., Paramedian pontine infarction. Neurological/topographical correlation. Stroke, 1997. **28**(4): p. 809-15.

9. Kim, J.S., Pure dysarthria, isolated facial paresis, or dysarthria-facial paresis syndrome. Stroke, 1994. **25**(10): p. 1994-1998.

10. Kim, J.S., Pure lateral medullary infarction: clinical–radiological correlation of 130 acute, consecutive patients. Brain, 2003. **126**(8): p. 1864-1872.

11. Kim, J.S. and J. Kim, Pure midbrain infarction: clinical, radiologic, and pathophysiologic findings. Neurology, 2005. **64**(7): p. 1227-32.

12. Kim, J.S., et al., Syndromes of pontine base infarction. A clinical-radiological correlation study. Stroke, 1995. **26**(6): p. 950-5.

13. Min, W.K., et al., Atherothrombotic cerebellar infarction: vascular lesion–MRI correlation of 31 cases. Stroke, 1999. **30**(11): p. 2376-2381.

14. Okuda, B., et al., Cerebral blood flow in pure dysarthria: Role of frontal cortical hypoperfusion. Stroke, 1999. **30**(1): p. 109-113.

15. Schmahmann, J.D., R. Ko, and J. MacMore, The human basis pontis: motor syndromes and topographic organization. Brain, 2004. **127**(6): p. 1269-1291.

16. Tanaka, K., et al., Pure dysarthria and dysarthria-facial paresis syndrome due to internal capsule and/or corona radiata infarction. BMC Neurology, 2015. **15**(1): p. 1-5.

17. Tohgi, H., et al., The side and somatotopical location of single small infarcts in the corona radiata and pontine base in relation to contralateral limb paresis and dysarthria. European neurology, 1996. **36**(6): p. 338-342.

18. Urban, P., et al., Dysarthria in acute ischemic stroke: lesion topography, clinicoradiologic correlation, and etiology. Neurology, 2001. **56**(8): p. 1021-1027.

19. Urban, P.P., et al., Impaired cortico-bulbar tract function in dysarthria due to hemispheric stroke. Functional testing using transcranial magnetic stimulation. Brain, 1997. **120**(6): p. 1077-1084.

20. Urban, P.P., et al., Cerebellar Speech Representation: Lesion Topography in Dysarthria as Derived From Cerebellar Ischemia and Functional Magnetic Resonance Imaging. Archives of Neurology, 2003. **60**(7): p. 965-972.

21. Urban, P.P., et al., Left-hemispheric dominance for articulation: A prospective study on acute ischaemic dysarthria at different localizations. Brain, 2006. **129**(3): p. 767-777.

22. Vuilleumier, P., J. Bogousslavsky, and F. Regli, Infarction of the lower brainstem. Clinical, aetiological and MRI-topographical correlations. Brain, 1995. **118 ( Pt 4)**: p. 1013-25.

23. Chung, C.-S., et al., Striatocapsular haemorrhage. Brain, 2000. **123**(9): p. 1850-1862.

24. Kim, D.H., et al., The pulvinar nucleus is associated with the presence of dysarthria in patients with basal ganglia hemorrhage. Neuroscience Letters, 2017. **655**: p. 131-136.
