# Supplementary material for: Neuroanatomical regions associated with non-progressive dysarthria post-stroke: a systematic review
Source: BMC Neurol. 2022 Sep 16;22:353. doi: 10.1186/s12883-022-02877-x (PMC9479301; doi:10.1186/s12883-022-02877-x)
Supplement: Supplementary file 2 — Additional file 2. [file 12883_2022_2877_MOESM2_ESM.docx]

**Neuroanatomical regions associated with non-progressive dysarthria post-stroke: A systematic review**

Marwa Summaka^1^, Salem Hannoun^2^, Hayat Harati^3^, Rama Daoud^4^, Hiba Zein^5^, Elias Estephan^3,6^, Ibrahim Naim^5^, Zeina Nasser^3*^

^1^Doctoral School of Sciences and Technology, Lebanese University, Hadath, Lebanon; ^2^Medical Imaging Sciences Program, Division of Health Professions, Faculty of Health Sciences, American University of Beirut, Beirut, Lebanon; ^3^Faculty of Medical Sciences, Neuroscience Research Center, Lebanese University, Hadath, Lebanon; ^4^Faculty of Medical Sciences, Lebanese University, Hadath, Lebanon ; ^5^Department of Rehabilitation, Health, Rehabilitation, Integration and Research Center (HRIR), Beirut, Lebanon; ^6^LBN Univ Montpellier, Montpellier, France.

Corresponding author: Zeina Nasser ^3^* MSc, PhD, Faculty of Medical Sciences, Neuroscience Research Center, Lebanese University, Hadath, Lebanon. Email: [z.nasser@ul.edu.lb](mailto:z.nasser@ul.edu.lb); ORCID: Zeinanasser2020; Telephone: 961 70950261-Fax: 961 1610920

**Table 1. The National Institutes of Health (NIH) quality assessment tool for cross-sectional and retrospective observational studies**

| Major Components | Response options | | |
| --- | --- | --- | --- |
| 1. Was the research question or objective in this paper clearly stated? | Yes | No | Cannot Determine/ Not Applicable/ Not Reported |
| 2. Was the study population clearly specified and defined? | Yes | No | Cannot Determine/ Not Applicable/ Not Reported |
| 3. Was the participation rate of eligible persons at least 50%? | Yes | No | Cannot Determine/ Not Applicable/ Not Reported |
| 4. Were all the subjects selected or recruited from the same or similar populations (including the same time period)? Were inclusion and exclusion criteria for being in the study prespecified and applied uniformly to all participants? | Yes | No | Cannot Determine/ Not Applicable/ Not Reported |
| 5. Was a sample size justification, power description, or variance and effect estimates provided? | Yes | No | Cannot Determine/ Not Applicable/ Not Reported |
| 6. For the analyses in this paper, were the exposure(s) of interest measured prior to the outcome(s) being measured? | Yes | No | Cannot Determine/ Not Applicable/ Not Reported |
| 7. Was the timeframe sufficient so that one could reasonably expect to see an association between exposure and outcome if it existed? | Yes | No | Cannot Determine/ Not Applicable/ Not Reported |
| 8. For exposures that can vary in amount or level, did the study examine different levels of the exposure as related to the outcome (e.g., categories of exposure, or exposure measured as continuous variable)? | Yes | No | Cannot Determine/ Not Applicable/ Not Reported |
| 9. Were the exposure measures (independent variables) clearly defined, valid, reliable, and implemented consistently across all study participants? | Yes | No | Cannot Determine/ Not Applicable/ Not Reported |
| 10. Was the exposure(s) assessed more than once over time? | Yes | No | Cannot Determine/ Not Applicable/ Not Reported |
| 11. Were the outcome measures (dependent variables) clearly defined, valid, reliable, and implemented consistently across all study participants? | Yes | No | Cannot Determine/ Not Applicable/ Not Reported |
| 12. Were the outcome assessors blinded to the exposure status of participants? | Yes | No | Cannot Determine/ Not Applicable/ Not Reported |
| 13. Was loss to follow-up after baseline 20% or less? | Yes | No | Cannot Determine/ Not Applicable/ Not Reported |
| 14. Were key potential confounding variables measured and adjusted statistically for their impact on the relationship between exposure(s) and outcome(s)? | Yes | No | Cannot Determine/ Not Applicable/ Not Reported |
| **Quality Rating** | Good | Fair | Poor |

**Table 2. The National Institutes of Health (NIH) quality assessment tool for case-series study**

|  | | | |
| --- | --- | --- | --- |
| Major Components | Response options | | |
| 1. Was the study question or objective clearly stated? | Yes | No | Cannot Determine/ Not Applicable/ Not Reported |
| 2. Was the study population clearly and fully described, including a case definition? | Yes | No | Cannot Determine/ Not Applicable/ Not Reported |
| 3. Were the cases consecutive? | Yes | No | Cannot Determine/ Not Applicable/ Not Reported |
| 4. Were the subjects comparable? | Yes | No | Cannot Determine/ Not Applicable/ Not Reported |
| 5. Was the intervention clearly described? | Yes | No | Cannot Determine/ Not Applicable/ Not Reported |
| 6. Were the outcome measures clearly defined, valid, reliable, and implemented consistently across all study participants? | Yes | No | Cannot Determine/ Not Applicable/ Not Reported |
| 7. Was the length of follow-up adequate? | Yes | No | Cannot Determine/ Not Applicable/ Not Reported |
| 8. Were the statistical methods well-described? | Yes | No | Cannot Determine/ Not Applicable/ Not Reported |
| 9. Were the results well-described? | Yes | No | Cannot Determine/ Not Applicable/ Not Reported |
| **Quality Rating** | Good | Fair | Poor |
